# i-Factor bone graft compared with allograft in spinal fusion for adolescent idiopathic scoliosis: a preliminary comparative study

**DOI:** 10.1097/BPB.0000000000001336

**Published:** 2026-02-16

**Authors:** Antti J. Saarinen, Aron Frantzén, Matti Ahonen, Ilkka Helenius

**Affiliations:** Departments of a Orthopaedics and Traumatology; b Paediatric Surgery, Orthopaedics and Traumatology, Turku University Hospital and University of Turku, Turku; c Department of Orthopaedics and Traumatology, University of Helsinki and Helsinki University Hospital; d Department of Paediatric Surgery, Orthopaedics and Traumatology, Helsinki New Children’s Hospital, Helsinki, Finland

**Keywords:** adolescent idiopathic scoliosis, allograft, amino-acid polypeptide, posterior spinal fusion

## Abstract

Bone graft extenders are widely used to supplement local autograft in posterior spinal fusion for adolescent idiopathic scoliosis (AIS). P-15, a novel synthetic 15-amino-acid polypeptide, has shown promise in promoting bone healing. This study compares P-15 (i-Factor) as a bone graft extender to allograft in adolescents undergoing segmental pedicle screw instrumentation for AIS. We conducted a retrospective analysis of prospectively collected data on 60 adolescents with AIS who underwent segmental pedicle screw instrumentation with a minimum 2-year follow-up. In this preliminary study, 30 patients received P-15, and 30 received an allograft in addition to a local autograft. Operative time, radiographic outcomes, and health-related quality of life were assessed. Plain radiographs were used to evaluate nonunion. Operative time was significantly shorter in the P-15 group (2.7 vs. 3.4 h; *P* = 0.01). At 2-year follow-up, the mean major curve was 17° (SD: 6.2) in the P-15 group and 20° (SD: 5.2) in the allograft group (*P* = 0.057). The mean loss of correction was similar between the groups (3.3 vs. 1.2°; *P* = 0.092). No cases of nonunion or deep surgical site infections were observed. Health-related quality of life scores were comparable between the groups. P-15 is, according to the current study, a safe and effective bone graft extender for posterior spinal fusion in adolescents with AIS, demonstrating similar radiographic and clinical outcomes compared to allograft while reducing operative time. Further research is warranted to assess the long-term results with P15 in adolescents undergoing pedicle screw instrumentation for idiopathic scoliosis.

## Introduction

Posterior spinal fusion (PSF) with pedicle screws has become a widely used corrective method for adolescent idiopathic scoliosis (AIS) [1,2]. Pedicle screw instrumentation allows better three-dimensional deformity correction when compared with prior methods [3]. PSF is performed using subperiosteal exposure, bilateral facetectomy, and bone grafting. Bone grafts are used on the decorticated bony elements. In AIS surgery, local bone autograft is typically augmented with either iliac crest autografts, allografts, or synthetic bone graft [4,5]. Iliac crest autograft is associated with longer operative time, increased blood loss, and postoperative pain. Allografts have shown a similar rate of fusion when compared with autografts in spinal surgery [4]. However, frozen allografts may have decreased osteoinductive properties. In addition, there is a risk of infectious disease transmission when allografts are used. Several synthetic grafts are being used in spinal surgery.

P-15 is a novel synthetic 15-amino-acid polypeptide that promotes bone healing and formation [6]. P-15 does not induce ossification in the surrounding soft tissue. i-Factor bone graft (i-Factor peptide-enhanced bone graft; Cerapedics Inc., Westminster, Colorado, USA) is a composite synthetic bone graft consisting of P-15 and bone mineral. i-Factor bone graft has been reported with a high rate of successful fusion in cervical and lumbar spinal surgery [6,7]. When compared with local autologous bone graft i-Factor has been shown to decrease the cost of treatment [6]. There are no previous comparative studies evaluating the use of i-Factor as a graft extender in adolescents undergoing surgery for idiopathic scoliosis.

We describe a matched patient series of adolescents treated operatively for idiopathic scoliosis with either i-Factor bone graft or allografts as a bone grafting method. We aimed to compare the intraoperative blood loss, radiological, and health-related quality of life outcomes to assess the safety and reliability of this synthetic bone graft extender as compared with allograft. We hypothesized that these outcomes would be similar in the study groups.

## Methods

### Patients

This is a preliminary retrospective study on prospectively collected data on adolescent patients who underwent operative treatment for AIS. The indication for PSF was a major curve of more than 45º in skeletally mature (Risser 4) adolescents. All patients treated with i-Factor bone graft with a minimum of 2 years of follow-up were included. These patients were matched using sex and age with patients treated with allografts. There were 30 eligible pairs. Institutional review board approval was obtained. Written consent was obtained from all participants.

### Surgical technique

All patients were operated by two senior pediatric orthopedic spine surgeons. Selection of bone graft was done chronologically, with patients treated after 2021 being operated with i-Factor and the allograft group between 2018 and 2021. Standardized operative technique with bilateral segmental pedicle screw instrumentation (Solera 5.5/6.0; Medtronic, MN, USA; MESA2; Stryker, Portage, MI, USA) was performed along with vertebral column derotation techniques with translation, compression, and distraction where needed. Placement of the screws was verified using intraoperative computed tomography (CT) imaging [8]. Selection of fusion levels was standardized according to the Lenke classification [9] with the last substantially touched vertebra as the lowest instrumented vertebra for Lenke 1 and 2 curves [10] and L3 or L4 for lumbar curves. We performed analysis at the following timepoints: preoperatively, after the initial surgery, at six months, and at 2-year follow-up.

### Radiological data

The major curve was measured using the Cobb technique from the coronal radiographs [11]. Thoracic kyphosis (T5–T12) and lumbar lordosis (T12–S1) were measured from the sagittal radiographs. Radiographs were evaluated for signs of nonunion, including halo formation around the fixation points, implant pull-out, screw breakage, or rod breakage.

### Health-related quality of life

The scoliosis society score 24 (SRS-24) is a disease-specific health-related quality of life questionnaire used to assess the current state of the patient with AIS and the effects of scoliosis surgery, consisting of 24 questions with a maximum score of 120 (5.0) [12]. The questionnaire has seven domains: pain, general self-image, function from back condition, general level of activity, postoperative self-image, postoperative function, and satisfaction. Each domain score ranges from 1 to 5, with higher scores indicating better patient outcomes.

### Statistical analysis

Statistical comparisons were performed with chi-squared tests for categorical parameters and *t* tests for continuous variables. Mann–Whitney *U* test was used to compare the SRS-24 domains between the study groups.

## Results

Clinical characteristics were similar between the groups (Table 1). Mean follow-up time was 2.6 years (2.0–6.0). The number of fused levels was similar between the groups. Operative time was significantly longer in the allograft group (*P* = 0.01). There was no significant difference in the intraoperative bleeding between the groups.

**Table 1:** Clinical characteristics of the groups

Variable	i-Factor (*n* = 30)	Allograft (*n* = 30)	*P* value
Age at surgery	15.6 (1.4)	15.7 (1.5)	0.933
Female	21 (70%)	21 (70%)	1.0
Levels fused	10.9 (2.1)	10.7 (1.9)	0.351
Operative time (h)	2.7 (0.8)	3.4 (1.2)	0.01
Intraoperative bleeding (ml)	696 (367)	811 (375)	0.116

Data are presented as means and SDs. *P* value was calculated using *t* tests.

Preoperative radiographic measurements were similar between the groups (Table 2). After the initial surgery, the mean major curve was 14° (SD: 5.0) in the i-Factor group and 19° (SD: 5.2) in the allograft group (*P* = 0.022). At the 6-month follow-up, the mean major curve was 17° (SD: 5.7) in the i-Factor group and 20° (SD: 5.6) in the allograft group (*P* = 0.017). At the 2-year follow-up, the mean major curve was 17° (SD: 6.2) in the i-Factor group and 20° (SD: 5.2) in the allograft group (*P* = 0.057). The mean loss of correction was similar between the groups (3.3 vs. 1.2°; *P* = 0.092). Thoracic kyphosis remained similar between the groups during the follow-up. Postoperative lordosis was 49° (SD: 13) in the i-Factor group and 43° (SD: 8.5) in the allograft group (*P* = 0.057). At the 6-month follow-up, the mean lordosis was 56° (SD: 11) in the i-Factor group and 46° (SD: 8.4) in the allograft group (*P* < 0.005). At the 2-year follow-up, the mean lordosis was 54° (SD: 13) in the i-Factor group and 47° (SD: 10) in the allograft group (*P* = 0.012).

**Table 2:** Radiological outcomes

Timepoint	i-Factor	Allograft	*P* value
Major curve			
Preoperative	49 (14.4)	44 (9.5)	0.602
Postoperative	14 (5.0)	19 (5.2)	0.022
6 Months	17 (5.7)	20 (5.6)	0.017
2 Years	17 (6.2)	20 (5.2)	0.057
Thoracic kyphosis			
Preoperative	15 (10)	18 (12)	0.273
Postoperative	21 (5.2)	21 (5.4)	0.882
6 Months	19 (5.2)	21 (5.7)	0.195
2 Years	23 (8.0)	25 (9.2)	0.274
Lumbar lordosis			
Preoperative	49 (14)	44 (9.5)	0.119
Postoperative	49 (13)	43 (8.5)	0.057
6 Months	56 (11)	46 (8.4)	<0.005
2 Years	54 (13)	47 (10)	0.012

Data are presented as means and SDs. *P* value was calculated using *t* tests.

There were no signs of nonunion in the study groups. In the i-Factor group, one patient experienced sensory loss in the groin area after the operation, one patient experienced seroma without infection, and one patient experienced postoperative ileus. In the control group, one patient required removal of malpositioned screws in the T10 vertebra, and one patient experienced persistent postoperative pain.

There were no differences in the SRS-24 domains between the groups at the 2-year follow-up (Table 3).

**Table 3:** SRS-24 scores at the 2-year follow-up

Domain	Allograft		i-Factor		
	Median	IQR	Median	IQR	Significance
SRS total score	4.2	0.5	4.1	0.8	0.609
Pain	4.7	0.9	4.4	0.8	0.397
Self image	4.3	0.4	4.2	1.1	0.962
Function	4.3	0.0	4.3	0.7	0.201
Activity	4.7	0.3	4.8	0.3	0.858
Postoperative self image	3.0	0.8	3.0	0.7	0.962
Postoperative function	3.0	0.3	3.0	1.0	0.448
Satisfaction	4.3	1.4	4.3	1.4	0.638

Data are presented as medians and IQR. *P* values were calculated using Mann–Whitney *U* test.

IQR, interquartile range; SRS-24, scoliosis society score 24.

## Discussion

i-Factor bone graft presented comparable radiographical results to allograft in patients who underwent PSF for AIS. The operative time was significantly lower in patients operated with the i-Factor bone graft. According to these preliminary results, i-Factor bone graft seems to be a safe method for PSF in AIS patients.

Allografts combined with local bone grafting have been routinely used in AIS surgery. Allografts help to avoid the problems with autografts, such as donor site bleeding, infection, and pain. However, there are concerns about the use of allografts. There is a small risk of transmission of infectious diseases when allografts are used. The quality of allograft bone may be suboptimal, as most of the grafts are harvested from femoral heads from hip replacement surgeries. We estimate that allograft preparation during the surgery increased the operative time, resulting to significant difference in the study groups. As the operative time increased, the intraoperative bleeding also tended to increase in the allograft group. However, given that the study is based on two consecutive patient series, we cannot exclude the influence of increasing surgical experience over time as a potential confounder.

i-Factor bone graft improves the bone ossification and fusion by the osteoinductive and osteogenetic properties. The P-15 peptide stimulates osteoblast differentiation in the bone marrow and cell adhesion to the fusion site. A previous study reported an increased level of successful fusion in noninstrumented lumbar fusion with P-15 when compared to allografts [13]. Another study reported a high rate of fusion in patients who underwent anterior lumbar fusion with i-Factor bone grafts [14]. Betz *et al*. [5] found no significant difference in outcomes between patients treated with an allograft and those without any grafting. However, long-term results from this approach remain scarce, and attributing fusion success solely to the graft substitute should be interpreted with caution. We hypothesize that the osteoinductive properties combined with the local bone graft led to similar rates of fusion, and no signs of nonunion were observed during a minimum 2-year radiographic follow-up.

## Limitations

This study had limitations. Our study design was a retrospective comparison on prospectively collected data on two consecutive cohorts. A post hoc power analysis indicated that a sample size of 30 patients per group would be sufficient to detect a clinically relevant 5° difference in loss of correction at 2-year follow-up (α = 0.05, power = 0.80). Both groups exceeded this threshold, suggesting that the analysis was adequately powered to detect such a difference. Surgical technique may have evolved during the data collection period. However, both cohorts underwent pedicle screw instrumentation for AIS with two experienced pediatric spinal surgeons with more than 10-year experience. Nonunion was evaluated with plain radiographs, while CT scans were not performed to limit radiographic exposure in teenagers. The intra- and interobserver variability of repeated coronal Cobb measurements has been reported to be 6° [15]. Based on this variability, we decided to define loss of correction as more than 5°. Screw placement was verified using intraoperative advanced imaging [8], and fusion levels were standardized according to the Lenke classification [9] with the last substantially touched vertebra as the lowest instrumented vertebra [10].

### Conclusion

Loss of correction was similarly low in patients treated with i-Factor synthetic bone graft and allografts at the 2-year follow-up. Operative time was significantly shorter in the i-Factor group. There were no signs of nonunion or deep surgical site infection in either cohort. Further research is warranted to assess the long-term results with P15, a synthetic 15-amino-acid polypeptide as the bone graft supplement in adolescents undergoing pedicle screw instrumentation for idiopathic scoliosis.

## Acknowledgements

I.H. has received research funding from Cerapedics and Medtronic. Dr. A.J.S. has received grants from the Päivikki and Sakari Sohlberg Foundation and Vappu Uuspää Foundation. A.F. has received grants from Finska Läkaresällskapet and Finnish Research Foundation for Orthopaedics and Traumatology.

### Conflicts of interest

There are no conflicts of interest.

## References

[1] de Kleuver M Lewis SJ Germscheid NM Kamper SJ Alanay A Berven SH. Optimal surgical care for adolescent idiopathic scoliosis: an international consensus. Eur Spine J 2014; 23:2603–2618. 24957258 10.1007/s00586-014-3356-1

[2] Crawford AH Lykissas MG Gao X Eismann E Anadio J. All-pedicle screw versus hybrid instrumentation in adolescent idiopathic scoliosis surgery: a comparative radiographical study with a minimum 2-year follow-up. Spine (Phila Pa 1976) 2013; 38:1199–1208. 23429683 10.1097/BRS.0b013e31828ce597

[3] Ledonio CG Polly DW Jr Vitale MG Wang Q Richards BS. Pediatric pedicle screws: comparative effectiveness and safety: a systematic literature review from the Scoliosis Research Society and the Pediatric Orthopaedic Society of North America task force. J Bone Joint Surg Am 2011; 93:1227–1234. 21776576 10.2106/JBJS.J.00678

[4] Price CT Connolly JF Carantzas AC Ilyas I. Comparison of bone grafts for posterior spinal fusion in adolescent idiopathic scoliosis. Spine (Phila Pa 1976) 2003; 28:793–798. 12698123

[5] Betz RR Petrizzo AM Kerner PJ Falatyn SP Clements DH Huss GK. Allograft versus no graft with a posterior multisegmented hook system for the treatment of idiopathic scoliosis. Spine (Phila Pa 1976) 2006; 31:121–127. 16418628 10.1097/01.brs.0000194771.49774.77

[6] Arnold PM Sasso RC Janssen ME Fehlings MG Heary RF Vaccaro AR Kopjar B. i-Factor™ bone graft versus autograft in anterior cervical discectomy and fusion: two-year follow-up of the randomized single-blinded food and drug administration investigational device exemption study. Spine J 2016; 16:S153–S154.

[7] Thaci B Yee R Kim K Vokshoor A Johnson JP Ament J. Cost-effectiveness of peptide enhanced bone graft i-Factor versus use of local autologous bone in anterior cervical discectomy and fusion surgery. Clinicoecon Outcomes Res 2021; 13:681–691. 34335035 10.2147/CEOR.S318589 PMC8318088

[8] Saarinen AJ Suominen EN Helenius L Syvänen J Raitio A Helenius I. Intraoperative 3D imaging reduces pedicle screw related complications and reoperations in adolescents undergoing posterior spinal fusion for idiopathic scoliosis: a retrospective study. Children (Basel) 2022; 9:1129. 36010020 10.3390/children9081129 PMC9406950

[9] Lenke LG Betz RR Harms J Bridwell KH Clements DH Lowe TG Blanke K. Adolescent idiopathic scoliosis: a new classification to determine extent of spinal arthrodesis. J Bone Joint Surg Am 2001; 83:1169–1181. 11507125

[10] Beauchamp EC Lenke LG Cerpa M Newton PO Kelly MP Blanke KM; Harms Study Group Investigators. Selecting the ‘touched vertebra’ as the lowest instrumented vertebra in patients with lenke type-1 and 2 curves: radiographic results after a minimum 5-year follow-up. J Bone Joint Surg Am 2020; 102:1966–1973. 32804885 10.2106/JBJS.19.01485

[11] Cobb JR. Outline for the study of scoliosis. Instr Course Lect 1948; 5:261–275.

[12] Haher TR Gorup JM Shin TM Homel P Merola AA Grogan DP. Results of the scoliosis research society instrument for evaluation of surgical outcome in adolescent idiopathic scoliosis: a multicenter study of 244 patients. Spine (Phila Pa 1976) 1999; 24:1435–1440. 10423788 10.1097/00007632-199907150-00008

[13] Jacobsen MK Andersen AK Jespersen AB. Randomized double blind clinical trial of ABM/P-15 versus allograft in noninstrumented lumbar fusion surgery. Spine J 2020; 20:677–684. 32001384 10.1016/j.spinee.2020.01.009

[14] Mobbs RJ Maharaj M Rao PJ. Clinical outcomes and fusion rates following anterior lumbar interbody fusion with bone graft substitute i-Factor, an anorganic bone matrix/P-15 composite. J Neurosurg Spine 2014; 21:867–876. 25325176 10.3171/2014.9.SPINE131151

[15] Morrissy RT Goldsmith GS Hall EC Kehl D Cowie GH. Measurement of the Cobb angle on radiographs of patients who have scoliosis: Evaluation of intrinsic error. J Bone Joint Surg Am 1990; 72:320–327. 2312527

